# Research progress in the pathogenesis of sepsis-associated encephalopathy

**DOI:** 10.1016/j.heliyon.2024.e33458

**Published:** 2024-06-22

**Authors:** Yue Zhou, Lu Bai, Wenjing Tang, Weiying Yang, Lichao Sun

**Affiliations:** a Teaching Department, First Hospital of Jilin University, Changchun, 130021, China; b Department of Medical Oncology, Dalian NO.3 People's Hospital, Dalian, 116091, China; c Department of Emergency Medicine, First Hospital of Jilin University, Changchun, 130021, China

**Keywords:** Sepsis, Sepsis-associated encephalopathy, Neuroinflammation, Epigenetics, Intestinal flora disorder, Hyperammonemia

## Abstract

Sepsis is a syndrome that causes dysfunction of multiple organs due to the host's uncontrolled response to infection and is a significant contributor to morbidity and mortality in intensive care units worldwide. Surviving patients are often left with acute brain injury and long-term cognitive impairment, known as sepsis-associated encephalopathy (SAE). In recent years, researchers have directed their focus towards the pathogenesis of SAE. However, due to the complexity of its development, there remains a lack of effective treatment measures that arise as a serious issue affecting the prognosis of sepsis patients. Further research on the possible causes of SAE aims to provide clinicians with potential therapeutic targets and help develop targeted prevention strategies. This paper aims to review recent research on the pathogenesis of SAE, in order to enhance our understanding of this syndrome.

## 1 Background

Sepsis, defined as a syndrome of multiorgan dysfunction caused by infection-induced dysregulation of the immune response, is a major cause of increased morbidity and mortality in intensive care units worldwide, resulting in approximately 6 million deaths per year [1]. When sepsis develops, the central nervous system may be affected first, manifested as diffuse brain dysfunction caused by systemic inflammation, secondary to internal infection rather than direct infection of the central nervous system, called sepsis-associated encephalopathy(SAE). SAE is a critical neurological syndrome characterized by diffuse brain dysfunction stemming from sepsis, which is a life-threatening condition due to dysregulation of the body's response to infection. As a major complication of sepsis, SAE is characterized by acute altered consciousness, including decreased attention, delirium, lethargy, coma, mood changes, long-term poor quality of life, and dementia.

Data suggest that sepsis survivors experience severe cognitive impairment, ICU-acquired weakness, and neuropsychiatric disorders after ICU discharge [2]. In randomized trials, one-third of adults with sepsis died after 6 months, and one-third were no longer able to perform activities of daily living [3]. Studies have also shown that SAE occurs in up to 70 % of patients with sepsis, with SAE having a mortality rate of 56.1 % [4]. Although SAE is considered a reversible syndrome, patients develop depression and long-term cognitive impairment after recovery from sepsis. In a longitudinal study of 1194 patients and 1520 hospitalized patients, the incidence of moderate to very severe neurologic deficit increased by 10.6 % and the incidence of moderate to very severe cognitive dysfunction increased threefold among sepsis survivors [5]. Cognitive dysfunction results from damage to the hippocampus and frontal lobes in early sepsis, while psychological disorders involve the limbic system [6].

The cognition of sepsis in patients and animal models began in the 1920s with the presence of inflammatory pathological changes in the brain that were initially attributed to the entry of microbial toxins into the brain [7]. In the early 2000s, attention turned to the effects of oxidative stress, cholinergic system, and cytokines on brain function after sepsis onset.There is no specific diagnostic criteria for SAE [8]. At present, exclusions are mainly adopted, that is, in the case that the patient has extracranial infection, the direct cerebral infection, metabolic encephalopathy, multiple organ failure, embolism, adverse drug reactions and other conditions are excluded, and the diagnosis of SAE is performed in combination with the patient's clinical symptoms and auxiliary examinations [9]. The scales commonly used to assess the mental status of SAE include Glasgow coma scale (GCS), confusion assessment method of intensive care unit(CAM-ICU), Montreal cognitive assessment (MoCA), Ramsay-Scale and Richmond agitation and sedation scale (RASS) [10]. Electroencephalography (EEG) is the most commonly used technique. As early as 1992, researchers found that EEG abnormalities were associated with the occurrence and severity of brain dysfunction in patients with sepsis. Early standard EEG abnormalities could also predict mortality in patients with sepsis [11]. Brain CT and MRI characteristic changes were closely related to SAE mortality and poor neurological prognosis [12]. Transcranial Doppler can also predict the development and prognosis of SAE.In addition, the exploration of the effect value of dynamic PET/CT imaging and multiparametric MRI, functional magnetic resonance imaging (fMRI) and proton magnetic resonance spectroscopy (1H-MRS), and ultrasound measurement of optic nerve sheath diameter (ONSD) in animal experiments of septic encephalopathy has also achieved certain results, but has not yet been promoted in clinical practice. However, prospective studies with large sample sizes are lacking, and there are currently no targeted therapies for early prevention and symptomatic treatment of SAE [13]. Therefore, as an extremely critical disease affecting the functional prognosis and life expectancy of patients with sepsis, it is important to clarify its mechanism and look for potentially modifiable factors affecting the prognosis of patients with SAE.

**Fig. 1:**
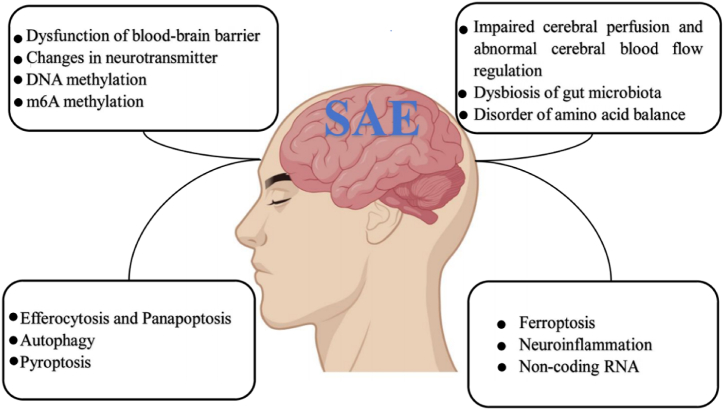
The pathogenesis of sepsis-associated encephalopathy.

Currently, most studies on SAE are exclusively based on animal experiments. Based on the existing research findings, the pathophysiological mechanism underlying SAE comprises numerous factors, such as impaired cerebral perfusion and abnormal regulation of cerebral blood flow, blood-brain barrier dysfunction, neurotransmitter dysfunction, neuroinflammation and cell death, epigenetic direction, intestinal flora imbalance, and amino acid imbalance. This paper aims to provide an overview of the research progress regarding the pathogenesis of SAE. The research progress of in the pathogenesis of SAE is shown in Fig. 1.

## 2 Methodology

### 2.1 Literature search strategy

The platforms searched for relevant literature were PubMed and Web of Science. The search terms used included “sepsis-associated encephalopathy,” “sepsis,” “epigenetics,”“inflammation,” “neuroinflammation,” and “immunology.” The search encompassed the literature published in the past 20 years. Additionally,a comprehensive review of the references of identified articles was conducted to identify additional relevant literature for inclusion.

### 2.2 Inclusion and exclusion criteria

This review included studies centred on the pathogenesis of sepsis-associated encephalopathy.It encompassed research elucidating the possible causes of SAE, which aims to provide clinicians with potential therapeutic targets and help develop targeted prevention strategies. Studies selected for inclusion were sourced from reputable journals and were included if the data was sufficient and relevant.

## 3 Impaired cerebral perfusion and abnormal cerebral blood flow regulation

Changes in cerebral perfusion play a crucial role in the pathology of SAE, leading to abnormal cell metabolism and oxidative stress. Compared to the control group, sepsis patients' cerebral blood flow (CBF) is significantly reduced, indicating a strong association with brain metabolic disorders [14]. Systemic hypotension, resulting in a decrease in cerebral perfusion, is common in sepsis. Ischemic injury was the most frequent morphological change in patients with SAE, and it manifested as purpura, perivascular edema, central pontine myelinolysis, multifocal necrotic leukoencephalopathy, astrocyte terminal paw swelling, and signs of neuronal apoptosis [6]. Microscopic studies of neurons revealed nucleus atrophy and cell membrane rupture. A prospective observational study found that 29 % of septic shock patients with SAE showed ischemic stroke on magnetic resonance imaging (MRI), and this was independently associated with disseminated intravascular coagulation and other factors [1]. Many brain regions are sensitive to ischemia and hypoxia, and the Amun horn of the hippocampus is particularly vulnerable, leading to changes in cognitive function. Studies have demonstrated that sepsis patients experience deficits in language learning and memory compared to non-sepsis patients, and their left hippocampus has significantly reduced volume, suggesting that hippocampal lesions are related to cognitive dysfunction [15]. Animal studies have shown that sepsis impairs cerebral microcirculation, resulting in decreased cerebral small blood vessel perfusion and functional capillary density in the sepsis group compared to the control group [16]. Impaired cerebral microcirculation in sepsis can result in cerebral hypoperfusion, which might be related to electrophysiological irregularities and neural function alterations. These metabolic and hemodynamic changes take place before any cognitive impairment or structural changes in the brain, such as white and gray matter atrophy.

Cerebral hemodynamic changes are a crucial aspect in the pathogenesis of SAE. Apart from systemic circulation disorders in sepsis patients, the self-regulation mechanism of cerebral blood flow is impaired, leading to varying degrees of abnormal perfusion. However, the mechanism behind abnormal cerebral blood flow regulation in SAE patients is not yet well-understood, and it could be related to the level of nitric oxide(NO). In normal physiological conditions cerebrovascular diastole is regulated by NO,whereas cerebrovascular contraction is controlled by endothelin [17]. In sepsis, the level of NO is influenced by inflammatory mediators, and the coupling between blood flow and metabolism,as well as the response of cerebrovascular to carbon dioxide, are disrupted, causing dysfunction in automatic regulation of the brain. Brain autoregulation is the process of maintaining steady-state cerebral blood flow (CBF) within the range of mean arterial pressure (MAP) of 60–160 mmHg. Sepsis triggers various mechanisms, including myogenic, neurogenic, metabolic, and endothelial, which can all be impaired, putting the brain at risk of global ischemia or cerebral edema. Studies have shown that reduced cerebral blood flow and impaired self-regulation in septic shock are associated with delirium.Studies have confirmed that sepsis patients with brain self-regulation change, blood flow and metabolism are out of coupling, and cerebrovascular response to CO_2_, is disordered, thereby aggravating brain tissue hypoxia and organ damage, and a series of neuropsychiatric symptoms such as delirium occur [18].

## 4 Dysfunction of the blood-brain barrier

The blood-brain barrier is a selective semi-permeable membrane that consists of a physical barrier (tight endothelial connections) and a transport barrier (receptor-mediated transcytosis) [19]. It plays a crucial role in maintaining the integrity of the brain and protecting its function by regulating ion concentration gradients and nutrient availability. The blood-brain barrier (BBB) regulates capillary blood flow in the brain and maintains the internal microenvironment to ensure effective neuronal function by controlling the transfer of nutrients, metabolites, and toxins [20]. When sepsis occurs, BBB may become more permeable due to complement activation, inflammatory cytokines, and overexpression of intercellular adhesion molecules (ICAM) in brain capillaries. This increased permeability allows activated white blood cells to enter the brain, further exacerbating the inflammatory process. Pro-inflammatory cytokines like TNF-α, IL-1β, and IL-6 are produced and intensified in response to endotoxins such as lipopolysaccharide(LPS), ROS, and NO [21]. These mediators can alter cell function and disrupt homeostasis in the internal environment, leading to increased permeability. Additionally, these mediators activate MMP-2, MMP-9, and MMP-8 in the blood-brain barrier, proteins that work by breaking down connections between cells that make up the brain barrier. The inflammatory cascades and oxidative stress caused by sepsis are responsible for structural changes in the blood-brain barrier. IL-1β is one of the major cytokines involved in this pathophysiology, which induces the activation and release of vascular endothelial growth factor A (VEGF-A) and thymidine phosphorylase (TYMP) by astrocytes. These proteins negatively regulate the expression of tight junction proteins in brain endothelial cells, thereby promoting the loss of blood-brain barrier integrity [22]. Astrocytes are known to play a complex role in the immune response of the brain. They can secrete TGF-β, a cytokine that has anti-inflammatory properties and is able to reduce the activation of microglia, which helps in delaying the onset of inflammation and promotes tissue renewal. However, astrocytes can also respond to IL-1β, a pro-inflammatory cytokine, by increasing the expression of mRNA and protein of inflammatory cytokines such as TNF-α and IL-6. Although this response is important in recruiting immune cells to the site of injury or infection, prolonged or excessive inflammation can lead to harmful effects such as neurodegeneration, astrocyte hyperplasia, and damage to the blood-brain barrier. Therefore, the immune response of astrocytes is intricate and context-dependent, with both protective and detrimental effects on the brain.

In an LPS-induced rat model of SAE, it was found that BBB dysfunction was associated with the condition [23]. Recent studies have shown that polymerase delta-activated protein 2 mediates LPS-induced changes in BBB permeability through the NF-κB/cyclooxygenase-2 signaling pathway. Brain imaging of SAE has revealed that in this condition, excess blood-brain barrier permeability causes tissue ischemia primarily in the white matter, which indicates a poor prognosis [24]. Sharshar et al. used MRI technology to evaluate SAE brain injury, confirming that BBB permeability increases in sepsis and is linked to poor prognosis [25]. Another animal model experiment demonstrated that tight junction protein levels were low in SAE, and mitochondrial dysfunction of microvascular endothelial cells was one of the mechanisms of BBB destruction [26].Studies have also shown that the increased permeability of BBB during sepsis leads to the activation of neuromicroglia and the production of cytotoxic mediators, which in turn act on BBB to further increase its permeability [27]. Taken together, these findings suggest that the destruction of BBB is both a cause and consequence of SAE.

## 5 Neurotransmitter changes

Neurotransmitter changes have long been believed to play a significant role in SAE development. Specifically, failure of cholinergic neurotransmission, particularly acetylcholine (Ach), has been shown to potentially underlie delirium and SAE symptoms. In sepsis, the vagus nerve can transmit inflammatory signals to the central nervous system, stimulating the release of acetylcholine from vagus nerve endings to regulate the inflammatory response. In SAE mice, studies have shown decreased expression of acetylcholine receptor in the hippocampus and inhibited functioning of the cholinergic anti-inflammatory pathway of the vagus nerve, resulting in uncontrolled inflammatory response and neurological dysfunction [28]. Research has also revealed that cholinergic depletion can lead to cognitive dysfunction induced by LPS, with rat models injected with LPS exhibiting behavioral changes and long-term memory deficits possibly linked to cholinergic dysfunction in the cerebral cortex, prefrontal cortex, and hippocampus. Notably, the administration of acetylcholinesterase inhibitors has been found to improve this dysfunction, thus upholding the theory that sepsis-related damage arises from cholinergic signaling dysfunction [29]. A study demonstrated that the application of selective α7 nAChR agonist PHA 568487 can significantly improve the nervous system's inflammatory response and memory dysfunction aggravated by LPS [30].

Brain-derived neurotrophic factor (BDNF) is a crucial element in hippocampal synaptic plasticity and cognitive function. Research has shown that sepsis animal models exhibit decreased BDNF levels in hippocampal tissue, leading to cognitive dysfunction and highlighting the significance of BDNF in SAE [31]. Inhalation of 2 % H2 has been found to relieve SAE in mice by controlling neurotrophic factors and genes linked to hippocampal plasticity via the Pparα-mediated CREB-BDNF signaling pathway [32]. Moreover, the density of gamma-aminobutyric acid type A receptors in the forebrain of sepsis rats increased, which may contribute to the pathogenesis of SAE dysfunction [33]. Inflammatory processes can trigger changes in numerous neurotransmitter systems, including glutaminergic, monoaminergic, and neurotrophic pathways, ultimately resulting in behavior changes. Riluzole's proven ability to inhibit glutamate release into synaptic space reduced the severity of neurological symptoms of experimental sepsis in rats and increased their survival. This finding implies the critical role of glutamate, neurotransmitter, and receptor expression disorders in SAE.

## 6 Neuroinflammation is associated with ferroptosis, pyrodeath, autophagy, exocytosis and panapoptosis

### 6.1 Neuroinflammation

Many studies have shown that neuroinflammation is a possible pathogenic mechanism of SAE combined with long-term cognitive impairment and is one of the main processes in which SAE is involved [34]. Activation of microglia and endothelial cells and BBB and astrocytes dysfunction are key components involved in neuroinflammation [35]. LPS can spread through peripheral inflammatory factors across the blood-brain barrier to the central nervous system, indirectly producing neuroinflammation, resulting in inflammatory damage to the central nervous system [11]. Microglia-activated immunophenotypes range from pro-inflammatory (M1)-releasing pro-inflammatory cytokines (such as γ-interferon or tumor necrosis factor-α) to anti-inflammatory (M2)-releasing immunomodulatory cytokines (such as IL-4 or IL-10). In sepsis, microglial activation is predominantly pro-inflammatory and is associated with neuronal apoptosis and disturbances of synapses and neurochemicals. Recent research concludes that the application of the colony stimulating factor 1 receptor (CSF1R) inhibitor PLX5622 may be sufficient to attenuate long-term neurocognitive dysfunction by reducing microglia-induced synaptic attachement/engulfment and preventing chronic microgliosis [36].They founds that microglia mediate neurocognitive deficits in SAE by eliminating C1q-tagged synapses.Therefore,complement-dependent synaptic pruning of microglia is an important pathological mechanism for the development of neuronal defects during SAE [37].One study suggested that activated microglia and complement cascade C1q signaling in hippocampus may be associated with synaptic loss and cognitive impairment in a mouse model of neuroinflammation induced by repeated injections of LPS [38].Studies have shown that inhibiting the activation of microglia can reduce neuroinflammation and oxidative damage to the brain, and improve long-term cognitive function in mice with sepsis, suggesting that microglia play an indispensable role in SAE [39]. Xu et al. found that RvD1 significantly inhibited the production of microglia TNF-α, IL-6 and IL-1β by weakening the activation of NF-κB, MAPKs and STAT signaling pathway molecules, and found that RvD1 had a protective effect on SAE mice, providing a new treatment for the treatment of SAE [40]. Studies have shown that Foxc1 overexpression improves SAE-induced cognitive dysfunction by regulating the IκBα/NF-κB pathway to inhibit microglial migration and neuroinflammation.Astrocytes are known to play a crucial role in coordinating the immune cells in the central nervous system by effectively monitoring and integrating inflammatory signals [41].

Astrocytes have been reported to coordinate the role of immune cells in the central nervous system through monitoring and integration of inflammatory signals. However, in cases of sepsis, abnormal astrocyte responses can lead to intractable neuroinflammation and cognitive impairment [42]. Endothelial cells also contribute significantly to brain degeneration following the onset of sepsis. This is due to their direct association with the breakdown of the blood-brain barrier, increased infiltration of inflammatory cells, abnormal microglia migration, and excessive formation of ROS and NO [43]. When endothelial cells are damaged by neuroinflammation, it leads to a brain perfusion disorder complicating the ischemic process of SAE. Result suggests that activating astrocytic α2A adrenoceptors in hippocampus can reduce glutamate toxicity specificially to attenuate SAE in mice.The cAMP/protein kinase A(PKA) signaling pathway may be a potential cellular mechanism, which can activate α2A-AR modulates astrocytic function [44]. If the integrity of the brain endothelial cells is altered, it can lead to the destruction of the BBB, thus increasing the permeability of peripheral neurotoxic factors. The combination of these factors further intensifies neuroinflammation, ultimately leading to the transformation of SAE into cognitive dysfunction.

### 6.2 Ferroptosis and neuroinflammation

Since Dixon et al. first proposed the concept of "ferroptosis" in 2012, it has been widely recognized as a mode of cell death that is driven by iron-dependent phospholipid peroxidation [45]. Ferroptosis is regulated by a variety of cellular metabolic pathways, including REDOX homeostasis, iron metabolism, mitochondrial activity, and metabolism of amino acids, lipids, and sugars, as well as various disease-related signaling pathways. The most typical features of ferroptosis are mitochondrial morphological changes. Wang et al. used transmission electron microscopy to confirm the existence of iron death in SAE. Using a cecal ligation and puncture (CLP)-induced sepsis mouse model, this study demonstrated that irisin protects against SAE by improving cognitive dysfunction, neurological deficits, and BBB disruption, and improving the inflammatory microenvironment through inhibition of hippocampal iron death via the Nrf2/GPX4 signaling pathway [46]. Chen et al. showed that sepsis induces high expression of exosomal derived NEAT1, which may aggravate SAE by promoting iron death through regulation of the miR-9-5p/TFRC and GOT1 axis [47]. In Xie et al.'s study, inhibition of Fer-1 on iron death was shown to alleviate glutamate excitotoxicity, maintain neural integrity, and ultimately protect cognitive function of SAE [48]. STING induces iron death of macrophages independent of cGAS and interferon, thereby promoting multiple organ damage induced by sepsis [49]. The researchers found that HET0016 could reduce STING-induced iron death in peripheral blood mononuclear cells of sepsis patients and reduce mortality in sepsis mouse models, making it a potential therapeutic target.

### 6.3 Pyroptosis and neuroinflammation

Pyroptosis is a recently discovered form of programmed cell death that involves the GsdMd-nt, the n-terminal of Gasdermin-D protein (GSDMD). GSDMD-NT can bind to lipids in the cell membrane and form pores, leading to cell lysis and the release of inflammatory mediators CLP such as IL-1β. These mediators cause rapid changes in the phenotype of microglia in the brain, which then produce their own inflammatory mediators. In an induced sepsis animal model, a study was conducted to inhibit the expression of GSDMD and its lysed form GsdMd-NT by administering the caspase-1 inhibitor VX765 as a neuroprotective therapy to reduce pyrodeath of brain cells during sepsis. Caspase-1 inhibitors prevented the expression of GSDMD and the cleavage of GSDMD-NT, which in turn reduced the pyroptosis of brain tissue [50]. They also reduced the expression of IL-1β, MCP-1, and TNF-α in serum and brain tissue, and prevented the destruction of the blood-brain barrier and ultrastructure damage caused by sepsis. The authors hypothesized that the inflammatory mediators circulating in the peripheral blood during sepsis enter the brain through the damaged blood-brain barrier and trigger pyroptosis of nervous tissue [51]. Following pyroptosis, microglia activation and neurological dysfunction occur, leading to cognitive impairment. Studies have shown that inhibiting caspase-1 can protect the ultrastructure of the brain, particularly the blood-brain barrier, and significantly reduce apoptosis and the release of inflammatory cytokines, thereby preserving cognitive function in CLP-induced experimental sepsis mice [1]. NLRP1, NLRP3, and AIM2 are involved in inflammasome-mediated pyrodeath, which leads to blood-brain barrier injury [52]. The NLRP3/Caspase-1 pathway-induced pyrodeath can result in cognitive impairment in SAE mouse models. Inhibiting NLRP3 using MCC950 or caspase-1 using Ac-YV AD-CMK can reduce mortality, reverse cognitive impairment, and salvage neuronal damage [53]. It was also shown that the BET protein inhibitor JQ1 can protect the hippocampal blood-brain barrier and neuronal damage by reducing neuroinflammation in mice induced by LPS injection through the inhibition of the typical inflammasome-dependent corticosis pathway, which is mediated by cleaved Caspase1/11 [54]. Therefore, JQ1 may be a promising target for SAE prevention.

### 6.4 Autophagy and neuroinflammation

Autophagy is a natural intracellular degradation system that regulates the breakdown of intracellular contents, including proteins, organelles, and lipids, in a lysosomal-dependent manner. It plays a pivotal role in cell survival through the removal of damaged organelles and protein deposits, as well as by providing additional energy. Autophagy was found to protect ATP7B knockout cells from copper-induced death [55]. Recent studies have revealed that some autophagy-related genes, such as Beclin1 and LC3, are involved in the regulation of phagocytosis [56]. Additionally, research conducted by Cui's team showed promising results. Furthermore, they discovered that the TRIM14-USP14-BRCC3 protein complex inhibits selective autophagy degradation of KDM4D by removing the ubiquitination modification of histone demethylase KDM4D, which affects the methylation modification of histone, promotes the expression of specific inflammatory cytokines and regulates the molecular mechanism of inflammatory response from the epigenetic level [57]. Recent research has also shown that rapamycin can alleviate cognitive dysfunction in SAE mice by promoting autophagy in hippocampal neurons [57]. Cui discovered that palmitylation-mediated autophagy-activated protein inhibits the activation of NLRP3 inflammatories, thereby preventing persistent inflammation [58]. The findings suggest that zDHHC12, an NLRP3 palmitoacylated S-acyltransferase, may be a potential therapeutic target for NLRP3 inflammator-related diseases. Research founds that AQP4 exacerbates cognitive impairment in SAE by inhibiting astrocyte autophagy mediated by Nav1.6. Thus,AQP4 knock out ameliorated cognitive dysfunction [59]. Additionally, Luo et al. found that SESN2 can protect the inflammatory response of hippocampal neurons in SAE mice and reduce brain injury and loss of learning and memory function by promoting ULK1-dependent neuronal autophagy [60]. Therefore, SESN2 may serve as a novel drug intervention strategy for SAE treatment.

### 6.5 Efferocytosis and neuroinflammation

Exocytosis is the process by which phagocytes recognize and eliminate aging and apoptotic cells [60]. Macrophages, microglia, and dendritic cells are the most common cells involved in cytoburial. Annexin A1 (AnxA1) is a protein secreted by phagocytes that plays a vital role in the resolution of inflammation by promoting cytoburial in these cells. Studies have shown that AnxA1 may be phosphorylated by signal transducer and activator of transcription 6 (STAT6), which in turn upregulates the expression of peroxisome proliferator-activated receptor γ (PPARγ)/CD36, leading to macrophages and microglia cells changing from M1 type to anti-inflammatory M2 type, enhancing intercellular functionality, and further facilitating the process of cellular burial [61,62].

MFG-E8 is a cellular bridging molecule that can reduce inflammation and improve sepsis and SAE by promoting cellular burial. A decrease in the expression of MFG-E8 was observed in the hippocampus of SAE mice. However, the behavioral performance of SAE mice improved after the lateral ventricular injection of recombinant MFG-E8, and the expression level of pro-inflammatory factors and apoptosis rate decreased in the hippocampus of SAE mice. Studies by Cai et al. showed that inhibition of VDAC1 could alleviate cognitive dysfunction in SAE mouse models induced by CLP by reducing mitochondrial autophagy and increasing cytoburial, providing potential value for SAE treatment [61]. Exocytosis is expected to be a potential new target for the study and treatment of SAE.

### 6.6 Panapoptosis and neuroinflammation

The findings indicate that in the SAE model of newborn rats, pyrodeath, apoptosis, and necrotic apoptosis (PANoptosis) occur simultaneously. Research conducted by Mu showed that PANoptosis occurred in cortical nerve cells of newborn SAE rats, with apoptosis and pyroptosis occurring earlier than necrotic apoptosis [62]. In the MAPK signaling pathway family, p38 MAPK, ERK, and JNK were activated.Among the three main members of the MAPK family, the p38 MAPK signaling pathway can regulate the three cell death modes of PANoptosis simultaneously. When p38 MAPK was inhibited, pyroptosis and apoptosis were inhibited, and necrotic apoptosis was activated. However, further inhibition of necrotic apoptosis, apoptosis, and pyroptosis resulted in their reactivation. The toll-like receptor 9 (TLR9), a protein molecule upstream of the p38 MAPK signaling pathway, was significantly increased in the cortex of SAE newborn rats. Inhibition of TLR9 resulted in the inhibition of PANoptosis, p38 MAPK, and ERK, with JNK not changing. Inhibition of TLR9 could improve the cortical injury and survival rate of SAE newborn rats. Therefore, it can be inferred that TLR9 regulates nerve cell PANoptosis through the p38 MAPK signaling pathway, highlighting its potential as a target for treating nerve injury in SAE. An innate immune system receptor, was also found to be activated, indicating that immune responses may be involved in the process of PANoptosis in SAE.

## 7 Epigenetic mechanisms

### 7.1 DNA methylation

DNA methylation is a significant epigenetic mechanism that can either stimulate or inhibit transcription of different genes, regulating various cellular functions by adding a methyl group to the cytosine C-5 site of dinucleotides via DNA methyltransferase. Changes in chromatin structure, DNA conformation, DNA stability, and the way DNA interacts with proteins can be caused by DNA methylation, which ultimately controls gene expression.Key inflammatory cytokines and transcription factors like interferon (IFN), interleukin, and recognition receptors are also regulated by DNA methylation. In research conducted by Yu et al., inhalation of 2 % H2 was shown to alleviate SAE by modulating BDNF promoter IV methylation mediated by DNMT1 and DNMT3a-specific DNA methyltransferase DNMT1 in the hippocampus of sepsis mice [63]. In the central nervous system, DNA methylation plays an essential role in maintaining cognitive function and is involved in the formation of synaptic plasticity and cognitive memory through gene regulation and other mechanisms [64]. Furthermore, preclinical studies have shown that DNA methylation inhibitors reduce inflammation and organ failure [65].

### 7.2 m6A methylation

m6A is a dynamic mRNA modification that regulates post-transcriptional protein expression at multiple levels. In recent years, various studies have indicated that RNA m6A epigenetics are involved in the development and regulation of various diseases. RNA methyltransferase, demethylase protein, and other main regulatory factors of RNA m6A have been gradually revealed in the human body. However, the relationship between these factors in SAE remains unstudied [66]. It has been found that the m6A reading protein YTHDF1's mitigation effect may be achieved through up-regulation of WWP1 to promote NLRP3 ubiquitination and inhibition of Caspase-1-dependent pyrodeath [67]. In the SAE group, a study showed that the expression levels of BDNF, NSE, S-100β, and ICAM-5 were significantly higher when compared to the non-SAE group [68]. The expression level of METTL3 was increased and the expression level of FTO decreased in the SAE group, while no significant difference was observed in the other m6A regulatory factors. Additionally, a study has reported that the heterogeneity of sepsis may be mainly caused by m6A RNA methylation [69].

### 7.3 Non-coding RNA (ncRNAs)

Non-coding RNAs (ncRNAs) are RNA molecules that do not have the potential to encode proteins. They account for the majority of RNA produced by mammalian genomes, amounting to about 98–99 % of the total RNA [70]. Among ncRNAs, microRNAs (miRNAs), long-chain non-coding RNAs (lncRNAs), and circRNAs are important regulatory RNA molecules that play crucial roles in almost all normal developmental cellular processes, as well as in various diseases, including those affecting the central nervous system. MiRNAs are endogenous non-coding RNAs that are smaller in size, ranging from 20 to 25 nucleotides, and they can cross the blood-brain barrier more easily compared to proteins. This makes them a more sensitive biomarker for sepsis-induced blood-brain barrier damage and/or encephalopathy. Studies have shown that the JAK2/STAT3 signaling pathway induces the expression of miR181b [71]. These findings strongly suggest that the JAK2/STA T3/miR-181b axis is a therapeutic target for the protection of sepsis-induced blood-brain barrier injury. Studies from transcriptome analysis have shown increased expression of miR-370-3p in SAE brains and suggest possible SAE biomarkers [72]. YY1, as an important transcription factor, promotes microglial M2 polarization by upregulating TREM-2 by interacting with the miR-130a-3p promoter to alleviate SAE [73]. MiRNA-494 may further regulate the activation of microglia in SAE by regulating mitochondrial function, providing basic research data for the development of new SAE therapeutics [74]. The expression of lncRNA Neat1 in neuronal cells of mice with sepsis is increased, it interacts directly with the hemoglobin subunit β (Hbb) to mediate the expression of PSD-95, regulating dendritic spine density associated with cognition and learning, Neat1 and Hbb may be potential diagnostic targets and therapeutic strategies for SAE [75].

### 7.4 Dysbiosis of gut microbiota

The normal intestinal flora of an organism is primarily composed of Firmicutes and Bacteroidetes, which significantly influence human health by playing an important role in the intestinal barrier. However, an imbalance of intestinal flora can lead to bacterial translocation by causing an increase in intestinal permeability and inducing mucosal immune dysfunction. Such abnormal changes in intestinal microbes are closely associated with brain diseases, such as cognitive dysfunction [76]. Studies have revealed that the number of symbiotic bacteria, such as Acinetobacter, Methanobrevibacter, and Syner-01, significantly increase in experimental groups with sepsis-associated encephalopathy. In contrast, the number of opportunistic microorganisms, like Anaerofilum, Catenibacterium, and Senegalimassilia, decrease, suggesting that the diversity and number of intestinal flora decrease in SAE patients [68]. The intestinal flora and the gut-brain axis play crucial roles in the development of nervous system diseases, influencing biological processes of brain functions, including apoptosis of nervous system cells, immunity, metabolism, and the blood-brain barrier [77]. Furthermore, the enteric-brain axis connects the central nervous system to the gastrointestinal tract, and intestinal flora influences the structure and functionality of the central nervous system through the hypothalamic-pituitary-adrenal axis (HPA) and the neuroimmune system. Additionally, intestinal flora can affect gene expression, regulate cerebrospinal fluid formation, and influence the structure and function of dendrites [78]. In animal experiments with sepsis-induced delirium, septic mice showed reduced microbial diversity and more severe neurocognitive impairment, including an increased number of seizures, while fecal microbiota transplantation (FMT) alleviated the deleterious neurological effects [79].

Based on current research, it has been demonstrated that intestinal flora can play a significant role in mitigating SAE by reducing inflammation in the central nervous system through the cholinergic anti-inflammatory system and the production of metabolites. In a rat model of SAE, studies have shown that transplantation of normal rat feces can effectively reduce the activation of cerebral cortical microglia and the release of pro-inflammatory factors in the hippocampus, thereby alleviating SAE symptoms [80]. Furthermore, it has been observed that the vagus nerve plays a role in this remission effect, as evidenced by a reduction in its effectiveness following vagus nerve amputation. In addition to fecal transplantation, intestinal flora metabolites such as short-chain fatty acids, which are neurotransmitters, have been found to be involved in neural activation and regulation of synaptic activity in the proximal neurons of the enteric nervous system. Such metabolites are also associated with various mental disorders [81]. Research also indicates that intestinal flora plays a crucial role in the susceptibility to SAE, as evidenced by a study which showed that butyrate, a metabolite produced by intestinal flora, can activate the GPR109A/Nrf2/HO-1 signaling pathway and potentially provide neuroprotection in sepsis [82]. In a study involving mice, Chen et al. induced sepsis through CLP and found that the intestinal flora of SAE resistant (SER) mice were less damaged than that of SAE susceptible (SES) mice. Furthermore, mice given postoperative fecal gavage from SES mice showed more pronounced neuroinflammation compared to those given fecal gavage from SER mice [83]. Importantly, the intestinal flora of SER mice was noted to be rich in indole-3-propionic acid (IPA), a neuroprotective metabolite that has shown promise in mitigating SAE in mice. These findings suggest that sepsis-induced intestinal flora disturbances play a vital role in mediating the susceptibility of CLP-induced sepsis mice to SAE. Additionally, the intestinal flora derivative IPA may hold potential as both a neuroprotective compound and a potential therapeutic agent to prevent neuroinflammation in SAE. Other studies have also provided valuable insights into the relationship between intestinal flora and neurological disorders. For example, research conducted by Sarkis K Mazmanian's group at Caltech showed that mice with neurodevelopmental disorders exhibited elevated levels of 4-ethylphenol-sulfate (4EPS), a metabolite associated with intestinal flora [84]. Follow-up studies by the same team found that this gut-derived metabolite can enter the brain and promote anxiety-like behavior in mice by influencing oligodendrocyte function and neuronal myelin patterns in the brain. These findings suggest that targeting intestinal flora and its associated metabolites may hold promise in improving neurological disorders such as anxiety.

## 8 Disorder of amino acid balance

When sepsis occurs, increased catabolism can cause an imbalance in plasma levels of aromatic and branched-chain amino acids (AAA and BCAA) in patients [85]. Aromatic amino acids, which are precursors to neurotransmitters, can easily infiltrate the central nervous system and studies have shown that elevated levels of AAA, along with higher APACHE II scores, are positively correlated with mortality in sepsis patients. In fact, high plasma levels of AAA can be independent predictors of mortality in such patients. In sepsis rats, decreased concentrations of dopamine, norepinephrine, and serotonin metabolites are observed in the brain. However, infusion of BCAA can help restore the balance. In one study, after the infusion of *E. coli* LPS, the plasma BCAA/AAA ratio decreased, which was mainly due to increased serum phenylalanine concentration and decreased serum valine and isoleucine concentrations [86]. Phenylalanine can serve as a potential neurotoxin that contributes to the production of pseudoneurotransmitters causing brain dysfunction in sepsis patients.

Ammonia is a metabolite produced during the breakdown of amino acids. Increased serum ammonia in patients with sepsis is associated with poor prognosis and studies have confirmed that sepsis is one of the main causes of hepatic encephalopathy [87]. Hyperammonemia in sepsis can be classified into two types: hepatic hyperammonemia due to liver dysfunction and non-hepatic hyperammonemia. In patients with sepsis, liver dysfunction can cause reduced ammonia clearance and increased blood ammonia levels. Additionally, factors such as increased intestinal permeability due to infection, increased intestinal ammonia production from gastrointestinal bleeding, and metabolism of amino acids from parenteral nutrition may contribute to elevated blood ammonia in sepsis patients. Comparing the responses of wild-type mice to bacterial LPS with those of hyperammonemic mice, previous studies have found that hyperammonemic mice display increased sensitivity to LPS. Therefore, it is speculated that hyperammonemic mice may alter the degree of inflammatory response and the body's sensitivity to such response. Additionally, elevated blood ammonia levels are found to cause dysfunction of neutrophils and macrophages, leading to impaired phagocytosis and potentially resulting in "sepsis-like" immune paralysis. Ammonia can also promote reactive oxygen species production, inhibit NO synthetase in endothelial cells, reduce NO synthesis, impair vasodilation function, affect tissue perfusion, and ultimately lead to cognitive impairment. Numan et al. discovered that sepsis patients with elevated ammonia levels had significantly longer hospital stays than those without elevated ammonia levels, indicating that elevated blood ammonia levels may serve as a novel biomarker for sepsis [88]. Alexandre et al. found that blood ammonia levels >100 μmol/L were linked to intracranial hypertension and higher mortality [89]. Retrospectively analyzing 465 SAE patients admitted to the ICU, Zhao et al. found that serum ammonia levels were linked to higher SOFA score and lactate level in SAE patients without liver failure [90]. SAPS II and Charlson scores were independent risk factors for death in SAE patients and could be used to assess their prognosis.

## 9 Treatment and management

Despite progress in the pathophysiology of SAE, no specific treatment has been found so far, and a multidisciplinary approach is needed for diagnosis and management. Timely identification and treatment of potential infections and effective control of systemic factors that can lead to secondary brain injury are critical, often including the administration of antibiotics and supportive measures such as liquid recovery and vasopressors [91]. In addition, factors leading to secondary brain injury must be controlled, including maintaining adequate oxygenation levels and blood pressure, addressing metabolic imbalances, and detecting/treating seizures.Throughout the ICU period, it is important to keep in mind the potential neurotoxic effects associated with specific drugs such as midazolam and cefepime, and to closely monitor the patient for non-convulsive seizures [92]. Several studies have demonstrated the role of dexmedetomidine, an adrenergic α2 receptor agonist, in reducing inflammation, alleviating immunosuppression, and improving brain function [93]. It is essential to systematically monitor serum concentrations of potentially neurotoxic drugs such as beta-lactam antibiotics, calcineurin inhibitors, and antifungals [94]. The potential effect of targeted neurocritical care in optimizing patient outcomes in the acute phase warrants further investigation.

## 10 Predictive models for SAE

As far as we know, there are no predictive models for diagnosing SAE before 2020. Yang et al. developed a nomogram to predict mortality in known SAE patients. This study is the first to establish a predictive standard map of SAE based on sociodemographic and clinical data from 2535 sepsis patients to enable individualized screening of SAE in patients with sepsis [95].There are studies that aim to identify early and potential risk factors for SAE through review analysis of large clinical databases, and to establish a comprehensive prediction model for SAE patients. This study found that clinically associated SAE-related risk factors, including age,and drugs including carbapenem antibiotics, rapid sequential evaluation of organ failure (qSOFA), H2-antagonist, quinolone antibiotics, steroids, diphenhydramine hydrochloride, midazolam,and heparin sodium injection had a significant impact on the development of SAE [96]. Satisfactory results have been obtained by establishing the nomogram of SAE individualized prediction for sepsis patients. It has good clinical practical value, which helps doctors to identify SAE patients in time, help doctors to take timely intervention measures, reduce the incidence of SAE and improve the prognosis of patients.The study suggests that the ML model can be used to assess the prognosis of SAE patients in the intensive care unit (ICU). Overall, the ML model alone was able to predict 30-day mortality in SAE patients [97].This helps in early screening of at-risk SAE patients.This is especially crucial because early treatment may improve neurocognitie outcome. Future research should focus on the long-term prognosis of SAE patients and the underlying mechanisms of SAE.

## 11 Directions for future research

Biomarkers may help to detect and monitor SAE by targeting different structures involved in pathophysiologic processes. It should be an early diagnosis as well as a reliable outcome assessment. Terminal propeptide of CNP(NT-proCNP), protein S100β(an astrocytic marker protein indicating blood brain barrier disruption and neuronal injury), and neuron-specific enolase(NSE) or neurofilament, are biomarkers of endothelial dysfunction, microglial activation, and brain injury with axonal damage, respectively [98,99]. One study showed that in patients with sepsis, neurofilament light (NfL) levels increased over time, while in patients without sepsis, serum NFL levels stabilized. Notably, SAE patients had significantly higher plasma NFL values, which correlated with the severity of SAE. Elevated plasma NFL levels were also associated with a poorer prognosis [98]. A prospective study found that Glial Fibrillary Acidic Protein (GFAP), as a protein expressed by astrocytes, was at higher concentrations in the serum of SAE patients [100]. Concentrations of serum microRNAs (mRNAs) is also considered as the diagnostic and prognostic biomarker of SAE, although the application in clinical practice is still limited [101]. Tau is also one of the markers of brain injury, with a sensitivity of 81.1 % and a specificity of 86.1 % in predicting 28-day mortality in patients with severe sepsis [102]. Currently, the treatment of SAE is limited. Therefore, more and more in-depth studies to find the key points between inflammatory response and brain dysfunction and block them, so as to target the treatment of SAE patients are the goals of future exploration.

## 12 Conclusion

The incidence and mortality rates of SAE are high, and even survivors often experience cognitive dysfunction, which has a significant impact on their long-term health. Therefore, timely and effective evaluation and treatment of SAE are necessary. While the detailed mechanisms of SAE have not been fully elucidated, they are believed to be complex and multifactorial, with various potential pathogenic factors contributing to its occurrence and development. These mechanisms are interdependent and synergistic, where the activation of one factor may lead to the activation of others, which could be the root cause of SAE. Although specific diagnostic methods and treatment measures for SAE are lacking, the search for biomarkers with higher sensitivity and specificity could assist in the realization of precision medicine, along with anti-neuroinflammation treatment. Furthermore, the strict management of systemic infection, sepsis, and systemic inflammatory response syndrome is crucial. These key factors may be the most valuable direction for subsequent relevant studies, as they have great significance in improving the current situation of clinical diagnosis and treatment of SAE.

## Data availability statement

No data was used for the research described in the article.

## Funding

The present study was supported by the grants from Project of Jilin Provincial Finance Department(JLSWSRCZX2023-60),10.13039/100007847Jilin Province Science and Technology Agency Project (20210101350JC).

## Ethics declarations

Not applicable.

## Ethics approval and consent to participate

Not applicable.

## Consent for publication

Not applicable.

## CRediT authorship contribution statement

**Yue Zhou:** Writing – original draft. **Lu Bai:** Writing – review & editing. **Wenjing Tang:** Writing – review & editing. **Weiying Yang:** Writing – review & editing. **Lichao Sun:** Supervision, Conceptualization.

## Declaration of competing interest

The authors declare that they have no known competing financial interests or personal relationships that could have appeared to influence the work reported in this paper.

## Abbreviations

(SAE): sepsis-associated encephalopathy
(CNS): central nervous system
(EEG): electroencephalogram
(ICAM-5): intercellular adhesion molecule-5
(S-100β): soluble protein-100β
(CBF): cerebral blood flow
(MRI): magnetic resonance imaging
(NO): nitric oxide
(CBF): cerebral blood flow
(MAP): mean arterial pressure
(BBB): blood-brain barrier
(ICAM): intercellular adhesion molecules
(VEGF-A): vascular endothelial growth factor A
(TYMP): thymidine phosphorylase
(Ach): acetylcholine
(GSDMD): Gasdermin-D protein
(LPS): lipopolysaccharide
(AnxA1): Annexin A1
(STAT6): signal transducer and activator of transcription 6
(PPARγ): peroxisome proliferator-activated receptor γ
(CLP): cecal ligation and puncture
(PANoptosis): pyrodeath, apoptosis, and necrotic apoptosis
(TLR9): toll-like receptor 9
(IFN): interferon
(ncRNAs): Non-coding RNAs
(miRNAs): microRNAs
(lncRNAs): long-chain non-coding RNAs
(Hbb): hemoglobin subunit β
(HPA): hypothalamic-pituitary-adrenal axis
(FMT): fecal microbiota transplantation
(SER): SAE resistant
(SES): SAE susceptible
(4EPS): 4-ethylphenol-sulfate
(AAA and BCAA): aromatic and branched-chain amino acids
(ONSD): optic nerve sheath diameter
(NfL): Neurofilament light
(GFAP): Glial Fibrillary Acidic Protein
